# Assessment of demographic and clinical characteristics on functional status and disability of patients with stroke

**DOI:** 10.17712/nsj.2016.4.20160212

**Published:** 2016-10

**Authors:** Derya Memis, Erkan Kozanoglu, Bayram Kelle, Mustafa K. Goncu

**Affiliations:** *From the Department of Physical Medicine and Rehabilitation (Memis), Adana Numune Education and Research Hospital, and the Department of Physical Medicine and Rehabilitation (Kozanoglu, Kelle, Goncu), Faculty of Medicine, Cukurova University, Adana, Turkey*

## Abstract

**Objective:**

To determine the effects of demographic and clinical characteristics on mobility, disability, and activities of daily life of patients with stroke.

**Methods:**

This cross-sectional clinical study was performed in the Department of Physical Medicine and Rehabilitation in Cukurova University Faculty of Medicine in Adana, Turkey, between February 2011 and December 2011. The study included 126 patients with stroke. The Brunnstrom recovery scale (BRS), functional ambulation classification scale (FACS), modified Barthel index (MBI), modified Rankin scale (MRS), and Rivermead mobility index (RMI) were used in the evaluation of the functional status of stroke patients. Correlations between each scale and parameters including age, etiology, and duration of hemiplegia were assessed.

**Results:**

The major etiology of stroke was found as ischemic (77%). Hypertension was a major risk factor in both genders (72% for males, 85% for females). Statistically significant differences were found between ischemic and hemorrhagic stroke patients regarding the RMI, MBI, BRS, and the FACS (*p*<0.001). Age had a poor negative correlation with the FACS and RMI.

**Conclusion:**

It is suggested that age is an important risk factor for the development of stroke, but it has no strong effect on functional status and disability in patients with stroke. The BRS, FACS, MBI, MRS, and RMI scales can be used in stroke patients whether they are under or over 65 years old in order to evaluate functional status and disability.

Stroke, which is a leading cause of disability may result in survival with permanent sequelae in physical, psychological, and social functions.^1^,^2^ More than half of stroke survivors are left with persistent sensorimotor sequelae.^3^ Cognitive and motor impairments may influence the recovery of functional status in stroke patients during rehabilitation. They extend the duration of rehabilitation and negatively affect the independence in daily activities and quality of life.^4^ The primary objective of post-stroke treatment is to improve the independence of stroke survivors. The ultimate goals for the rehabilitation of stroke patients are to provide a functional independence necessary for daily activities, and to integrate them into community life.^5^ The risk factors are not fully understood,^6^ but are thought to be similar throughout the world.^7^ Studies investigating the relationship between age and quality of life in stroke patients have conflicting results.^2^ In addition, it is known that comorbid diseases and cognitive impairment have a negative effect on functional status.^8^ There are only a limited number of studies in the literature that aim to analyze the relationship between demographic and clinical characteristics and disability with functional status in patients with stroke. The aim of this study was to determine the effects of demographic and clinical characteristics on mobility, disability, and activities of daily life of patients with stroke.

## Methods

### Study design

This cross-sectional clinical study was performed in the Department of Physical Medicine and Rehabilitation in Cukurova University Faculty of Medicine, Adana, Turkey between February 2011 and December 2011. The Institutional Review Board of Cukurova University approved the study protocol. The Declaration of Helsinki protocols were followed, and the patients provided written informed consent. All subjects underwent a detailed systemic physical examination including neurologic and musculoskeletal evaluations. Hemiplegia was diagnosed according to the clinical history, neurological examination, and imaging via CT, or MRI of the brain.

### Inclusion criteria

Inclusion criteria were as follows: (1) >18 years of age, (2) unilateral hemiplegia, (3) stable medical condition, (4) sufficient cognitive ability to consent to the examination and treatment, (5) capable of independent activities of daily life before stroke.

### Exclusion criteria

Exclusion criteria were as follows: (1) bilateral hemiplegia, (2) hemiplegia secondary to malignancy or trauma, (3) pathologic cerebellar findings, (4) severe cognitive impairment.

The demographic data of patients and clinical characteristics including etiology, risk factors, and comorbidity were recorded. The Brunnstrom recovery scale (BRS) for functional recovery, functional ambulation classification scale (FACS) for ambulation level, modified Barthel Index (MBI) for activities of daily life, modified Rankin scale (MRS) for disability, and Rivermead mobility index (RMI) for the mobility level were used in the assessment. Patients were divided into 2 groups by age (<65 years or ≥ 65 years old). Correlations between scores in each group were recorded. The correlation of these parameters was assessed together with etiology (ischemic or hemorrhagic) and duration of hemiplegia (less than one year or more than one year), and whether the patient received a rehabilitation treatment program or not. The Brunnstrom scale was used for the evaluation of functional recovery of patients with stroke. The BRS includes 6 stages from I (flaccid limbs without any voluntary movement) to VI (well-coordinated movements).^9^ Spasticity was evaluated using the 5-point Ashworth scale from 0 (no increase in muscle tone) to 4 (rigid affected limb).^10^,^11^ The ambulation level was evaluated by the FACS, which was developed by the Massachusetts General Hospital and is used for the evaluation of patients’ level of ambulation. This scale includes 6 functional levels ranging from 0 (absence of walking capacity) to 5 (independent ambulation).^12^ The MBI, which has a Turkish adaptation is composed of 10 items with varying weights (2 items for personal care; 6 items for use of stairs, feeding, controlling of bowel and bladder; 2 items for walking and moving from the wheelchair). The MBI is a cumulative score calculated by summing each item with a range of 0 (completely dependent) to 100 (independent in basic activities of daily living [ADL]). Higher scores represent a higher degree of independence.^13^-^15^ The MRS consists of 6 levels for disability. Level 0 defines no symptom at all, and level 6 defines death.^16^,^17^ The RMI, which is a rating scale measuring the mobility including 15 items. Total scores are achieved by interviewing the patients with item scores summed to give a total score that ranges from 0 (all ‘no’ responses to items) to 15 (all ‘yes’ responses to items).^18^

### Statistical analysis

Statistical analysis was performed using STATA software (Statacorp LP, College Station, TX, USA). Quantitative variables were expressed as mean ± standard deviation and range. Student’s t test was used for the analysis of intergroup parametric data. Pearson correlation test was used for the analysis between parametric data. The percentage was calculated by Pearson’s Chi-square test. The limit of statistical significance was *p*<0.05.

## Results

A total of 148 patients with stroke were eligible for this study, however, 22 patients were excluded as they did not meet the inclusion criteria, and a total of 126 patients were included in the study. The clinical and demographic data are shown in **Table 1**. The mean age of the patients was 60.4 years. Right lateralization of stroke was 52% (n=66), and left lateralization was 48% (n=60). The major etiology of stroke was found as ischemic (77%). Hypertension was a major risk factor in both genders (72% for males, 85% for females); however, diabetes mellitus (39%) and hyperlipidemia (36%) were major risk factors in women, and hyperlipidemia (49%) and smoking (40%) were major risk factors for stroke in men (**Table 1**). Statistically significant differences were found between ischemic and hemorrhagic stroke patients regarding the RMI (*p*=0.001), MBI (*p*=0.005), BRS (upper extremity *p*=0.001; hand *p*=0.001, lower extremity *p*=0.001), and FACS (*p*<0.001). However, a statistically significant difference was not found using the MRS in patients with ischemic and hemorrhagic stroke (*p*=0.100) (**Table 2**). Correlations between the FACS, MRI, RMI, and MBI are shown in **Table 3**. Age had a poor negative correlation with the FACS (r=-0.075) and RMI (r=-0.096), but had a poor positive correlation with the MRS (r=+0.098). The RMI had a strong negative correlation with the MRS (r=-0.915), and the MRS had a strong negative correlation with the MBI (r=-0.905). Age was found as a poor indicator for functionality of stroke patients (**Table 3**). Patients who did not receive a rehabilitation program for stroke, and who had a duration of disease more or less than one year were divided into 2 arms as <65 years or ≥65 years old age. Statistically significant differences were not found regarding the RMI, MBI, MRS, FACS, and BRS both in patients who had a disease duration of more or less than one year (*p*>0.05, **Table 4**). Statistically significant differences were not found in patients who received any rehabilitation program with a disease duration more or less than one year (*p*>0.05, **Table 5**).

**Table 1:** Demographic and clinical characteristics of stroke patients and the distribution of risk factors for stroke according to gender (n=126).

Characteristics	Male		Female	
	n (%)			
Gender	65	(52)	61	(48)
Mean age (years)	58.9		61.2	
*Risk factors*				
Diabetes mellitus	20	(31)	24	(39)
Hypertension	47	(72)	52	(85)
Ischemic heart disease	12	(18)	6	(10)
Congestive heart failure	12	(18)	6	(10)
Myocardial infarction	3	(5)	-	-
Hyperlipidemia	32	(49)	22	(36)
Arrhythmia	3	(5)	-	-
Coronary artery disease	8	(12)	1	(2)
Smoking	26	(40)	7	(11)
Alcohol consumption	6	(9)	0	(0)
History of TIA	16	(25)	6	(10)
*Etiology of stroke (ischemic/hemorrhagic)*	>65 years		<65 years	
Distribution by age of ischemic patients	33	(34)	64	(66)
Distribution by age of hemorrhagic patients	9	(31)	20	(69)

TIA – transient ischemic attack

**Table 2:** Comparison of outcome measures according to the etiology of stroke.

Outcome measures	Ischemic (n=97)	Hemorrhagic (n=29)	*P*-value
	Mean ± standard deviation (min-max)	
RMI	8.31±4.63(0-15)	5.38±4.42(0-14)	0.001
MBI	60.66±27.6(0-100)	45.41±27.84(0-94)	0.005
MRS	2.85±1.16(1-5)	3.72±1.03(2-5)	0.100
FACS	2.87±1.61(0-5)	1.65±1.58(0-4)	<0.001
BRS					
Upper extremity	3.40±1.63(1-6)	2.41±1.23(1-5)	0.001
Hand	3.20±1.71(1-6)	2.17±1.25(1-5)	0.001
Lower extremity	3.79±1.25(1-6)	3.10±1.31(1-5)	0.001
MAS					
Upper extremity	0.62±0.85(0-3)	0.90±0.72(0-3)	0.942
Lower extremity	0.32±0.31(0-2)	0.62±0.86(0-3)	0.988

RMI - Rivermead mobility index, MBI - modified Barthel index, MRS - modified Rankın scale, FACS - functional ambulation classification scale, BRS - Brunnstrom recovery stage, MAS - modified Ashworth Scale

**Table 3:** Correlations between age, FACS, RMI, MRS and MBI among stroke patients.

Variable	FACS(r)	RMI(r)	MRS(r)	MBI(r)
Age	-0.075	-0.096	0.098	-0.126
RMI	0.918			
MRS	-0.885	-0.915		
MBI	0.900	0.917	-0.905	

RMI - Rivermead mobility index, MBI - modified Barthel index, MRS - modified Rankın scale, FACS - functional ambulation classification scale, BRS - Brunnstrom recovery stage, MAS - modified Ashworth Scale, r=-1/+1

**Table 4:** Comparison of outcome measures in stroke patients with disease duration more and less than one year, and who do not attend a rehabilitation program.

Variable	Patients <65 years		Patients ≥65 years		*P*-value
	Mean ± standard deviation (min-max)			
*>1 year*	**n=20**		**n=6**		
RMI	7.9±5.09	(0-15)	9.67±5.82	(2-15)	0.762
MRS	3.15±1.14	(1-15)	2.33±1.51	(1-4)	0.082
MBI	55.8±30.57	(2-100)	61.33±45.78	(4-100)	0.634
FACS	2.3±1.63	(0-5)	3.17±2.23	(0-5)	0.849
BRS – upper ext	2.9±1.71	(1-6)	3.33±1.97	(1-5)	0.698
BRS – lower ext	3.45±1.37	(1-5)	4±1.26	(2-5)	0.807
BRS – hand	2.75±1.80	(1-6)	3.33±1.97	(1-5)	0.749
*<1 year*	**n=28**		**n=14**		
RMI	7.57±5.17	(0-15)	6.36±4.77	(0-14)	0.233
MRS	2.93±1.33	(1-5)	3.29±1.33	(1-5)	0.296
MBI	58.04±29.52	(0-100)	52.71±31.25	(1-100)	0.791
FACS	2.5±1.86	(0-5)	2.29±1.77	(0-5)	0.361
BRS – upper ext	3.29±1.84	(1-6)	3.93±1.73	(0-5)	0.858
BRS – lower ext	3.7±1.51	(1-6)	4±1.47	(0-5)	0.180
BRS – hand	3.25±1.88	(1-6)	3.86±1.83	(0-5)	0.837

RMI - Rivermead mobility index, MRS - modified Rankın scale, MBI - modified Barthel index, FACS - functional ambulation classification scale, BRS - Brunnstrom recovery stage, ext - extremity

**Table 5:** Comparison of outcome measures in stroke patients with disease duration more and less than one year, and attended a rehabilitation program.

Variable	Less than one year (n=18)		Less than one year (n=40)		*P*-value
	Mean ± standard deviation (min-max)			
RMI	6.44±4.85	(0-15)	8.23±4.03	(0-15)	0.925
MRS	3.39±1.20	(1-15)	2.95±1.01	(1-5)	0.947
MBI	49.94±28.73	(0-100)	61.38±22.36	(23-100)	0.077
FACS	2.22±1.73	(0-5)	2.98±1.42	(0-5)	0.956
BRS – upper ext	2.67±1.50	(1-6)	3.18±1.26	(1-6)	0.908
BRS – lower ext	3.44±1.28	(1-5)	3.58±1.11	(1-6)	0.654
BRS – hand	2.61±1.50	(1-6)	2.75±1.37	(1-6)	0.634

RMI - Rivermead mobility index, MRS - modified Rankın scale, MBI - modified Barthel index, FACS - functional ambulation classification scale, BRS - Brunnstrom recovery stage, ext - extremity

## Discussion

Stroke which is one of the most important health problems resulting in major disability and death. Life satisfaction and quality of life of stroke survivors are significantly lower than of the general population. The most important point for the prevention of stroke is the determination of people with higher risk factors for stroke, and protecting them from this catastrophic event. Nowadays, most stroke risk factors have been defined, and are reported as curable.^19^ Age is the most important risk factor for stroke, and the risk is doubled beyond the age of 55.^20^ In the present study, the mean of age of the patients was 64 years, and this result is consistent with the literature. However, age did not have a major effect on functional status in our study. Although age is one of the unmodifiable risk factors, there are modifiable risk factors including hypertension, diabetes mellitus, smoking, and so forth. Cabral et al^21^ and Arboix et al^22^ reported that hypertension was the major risk factor for cardiovascular diseases. The results of the present study were consistent with the previous studies. Hypertension was also found as a major risk factor in both genders. Interestingly, it was found that smoking was not a significant risk factor for female patients suffering from stroke, while it was found as an important risk factor for males. This condition may be explained by the higher smoking rates in males in our country.

Because of the distinction by the means of prognosis and incidence rates, the etiology of stroke is important. Numerous studies have demonstrated that ischemic stroke is more frequent.^23^,^24^ In our study, the number of patients with ischemic stroke was higher than patients with hemorrhagic stroke, consistent with the current literature. It was reported that elderly patients (over 85 years) with lacunar stroke had greater focal neurological impairment.^25^ This neurological impairment may lead to lower functional status. However, lacunar stroke may cause this poor status or the disability may be related to comorbidities found in the elderly period. We did not evaluate the elderly patients who were 85 years and older.

Approximately 50% improvement in functional status is expected one year after stroke. However, it has been reported that the prognosis is not clear in post stroke patients with a disease duration less than one year. The risk of mortality is higher, and these patients do not have the same therapeutic goals as patients with a disease duration longer than one year.^26^ The concept of ‘‘quality of life’’ is complex and there is no unanimously accepted definition.^2^ It depends on social and cultural backgrounds, and and it changes according to the countries.

The relationship between MRS and MBI was evaluated in a previous study.^5^ It was reported that these 2 parameters were associated in the early disease period, and the maximum sensitivity of MRS is reached at 6 months post-stroke.^5^ In the present study, we found a prominent negative correlation between these 2 parameters. In another multicenter study,^27^ it was suggested that the MRS is used as a subjective global disability scale, which measures changes in activity and lifestyle by the time after stroke that does not always match the basic ADL measured by the MBI.^27^ This study was cross-sectional, so changes on the MRS and MBI by time could not be evaluated.

Chou et al^28^ found a moderate correlation between the MBI and RMI. Authors reported that a dynamic balance was measured using the RMI in the evaluation of falls. There was a strong positive correlation between MBI and RMI in our study. In the aforementioned study,^28^ because of the lack of separate BRS scores it is difficult to discuss the effects of lower extremity BRS values on falls. Pure motor stroke was reported as the most common result of lacunar infarct, but the prognosis was found better than other stroke syndromes.^29^ Mobility in stroke patients has generally been evaluated with the RMI, MBI, and FACS, and a positive correlation between these scores has been reported.^30^,^31^ Improved motor activity measured by BRS, and improved mobility measured by RMI after balance training have also been reported. At the end of the rehabilitation process, improvements in these parameters have been reported.^32^ In the current study, patients that did not participate in a rehabilitation program were grouped according to disease duration and no difference was found in the RMI, MBI, MRS, FACS, and BRS scores. Nevertheless, the results of patients who received a rehabilitation program were similar to the patients who did not receive a rehabilitation program. This condition can be attributed to the small number of patients, and to the lack of a longer follow-up period.

There were some limitations to the current study. The number of patients was relatively small, and the study was designed as cross-sectional. Therefore, we could not able to demonstrate the changes of parameters by time. Because of a specific rehabilitation treatment program was not applied, we could not evaluate the effects of a rehabilitation program on functional parameters in patients with stroke.

In conclusion, it is suggested that age is an important risk factor for the development of stroke, but it has no strong effect on functional status and disability in patients with stroke. The ischemic stroke patients had better results for functional status. The BRS, FACS, MBI, MRS, and RMI scales can be used in stroke patients whether they are under or over 65 years old in order to evaluate the functional status and disability. Further studies are needed using different functional indexes in patients with stroke who have undergone rehabilitation program in the evaluation of the effects of therapy on functional status and disability.
